# COVID-19 Transcriptomic Atlas: A Comprehensive Analysis of COVID-19 Related Transcriptomics Datasets

**DOI:** 10.3389/fgene.2021.755222

**Published:** 2021-12-22

**Authors:** Fatma Alqutami, Abiola Senok, Mahmood Hachim

**Affiliations:** ^1^ College of Medicine, Mohammed Bin Rashid University of Medicine and Health Sciences, Dubai, United Arab Emirates; ^2^ Center for Genomic Discovery, Mohammed Bin Rashid University of Medicine and Health Sciences, Dubai, United Arab Emirates

**Keywords:** COVID-19, SARS – CoV – 2, omics analyses, differentially expressed gene analysis, atlas

## Abstract

**Background:** To develop anti-viral drugs and vaccines, it is crucial to understand the molecular basis and pathology of COVID-19. An increase in research output is required to generate data and results at a faster rate, therefore bioinformatics plays a crucial role in COVID-19 research. There is an abundance of transcriptomic data from studies carried out on COVID-19, however, their use is limited by the confounding factors pertaining to each study. The reanalysis of all these datasets in a unified approach should help in understanding the molecular basis of COVID-19. This should allow for the identification of COVID-19 biomarkers expressed in patients and the presence of markers specific to disease severity and condition.

**Aim:** In this study, we aim to use the multiple publicly available transcriptomic datasets retrieved from the Gene Expression Omnibus (GEO) database to identify consistently differential expressed genes in different tissues and clinical settings.

**Materials and Methods:** A list of datasets was generated from NCBI’s GEO using the GEOmetadb package through R software. Search keywords included SARS-COV-2 and COVID-19. Datasets in human tissues containing more than ten samples were selected for this study. Differentially expressed genes (DEGs) in each dataset were identified. Then the common DEGs between different datasets, conditions, tissues and clinical settings were shortlisted.

**Results:** Using a unified approach, we were able to identify common DEGs based on the disease conditions, samples source and clinical settings. For each indication, a different set of genes have been identified, revealing that a multitude of factors play a role in the level of gene expression.

**Conclusion:** Unified reanalysis of publically available transcriptomic data showed promising potential in identifying core targets that can explain the molecular pathology and be used as biomarkers for COVID-19.

## Introduction

The global pandemic COVID-19 is caused by the novel coronavirus SARS-CoV-2 and has infected over 110 million people, resulting in over 2.4 million deaths worldwide (WHO, 2020). The initial outbreak began in a Seafood Wholesale Market in Wuhan, China, in December 2019 and has spread across the world (Bai et al., 2020). It has been suggested that SARS-CoV-2 is transmitted via respiratory droplets, surface contamination, and aerosols, though the latter’s significance is unclear (Asadi et al., 2019; Wiersinga et al., 2020).

COVID-19 causes many symptoms with most common features including: fever, cough, fatigue, and shortness of breath (Huang et al., 2020). Lesser-known symptoms include headaches, diarrhea, and hemoptysis (Huang et al., 2020). The severity of the disease progresses from the initial onset of the first symptom, causing a deterioration in the patient’s health, followed by the development of acute respiratory distress syndrome (ARDS) and accompanied by ICU admission in a short time period (Guan et al., 2020; Huang et al., 2020). The heterogeneity of COVID-19 results in the differing onsets and severity of symptoms between patients, therefore, making it challenging to identify disease-specific biomarkers.

SARS-CoV-2 is classified as an enveloped, positive-sense, single-stranded RNA β coronavirus (Channappanavar et al., 2014). Similarly to severe acute respiratory syndrome coronavirus (SARS-CoV) and Middle East respiratory syndrome coronavirus (MERS-CoV), SARS-CoV-2 and other β coronaviruses are linked to respiratory infections in a host of species (Channappanavar et al., 2014; Yuki et al., 2020).

The viral life cycle of SARS-CoV-2 begins when the virus’ Spike (S) protein attaches to the host cell’s surface receptor (Bosch et al., 2003; Yuki et al., 2020). The cellular receptor Angiotensin-converting enzyme 2 (ACE2) has been identified as the receptor facilitating this attachment (Li et al., 2003; Chen et al., 2020; Letko et al., 2020; Walls et al., 2020). Once the virus adheres to the cell surface, a variety of proteases including the transmembrane protease serine 2 (TMPRSS2) cleaves the S1 and S2 subunits of the S protein which allows for cellular entry (Hoffmann et al., 2020; Ou et al., 2020; Yuki et al., 2020). Studies have shown that ACE2 and TMPRSS2 are highly expressed in lung epithelial cells, which coincides with the entry route of the virus (Shu et al., 2020; Yuki et al., 2020). Upon cellular entry, the virus replicates and matures before releasing itself into the host body (Yuki et al., 2020). It takes approximately 5 days for symptoms to develop following the initial exposure to the virus due to the host mounting an active immune response ((Lauer et al., 2020; Wiersinga et al., 2020).

Following the initial exposure, the host immune system activates its viral inflammatory response. The innate airway immune response includes epithelial cells, alveolar macrophages, and dendritic cells (DCs) (Yoshikawa et al., 2009; Yuki et al., 2020). These antigen-presenting cells present the S protein to CD4^+^ T cells, activating B cells as part of this response (Yuki et al., 2020). During the initial stages of the disease there is an increase in IgM and IgA antibodies, followed by a prolonged presence of IgG (García, 2020). In severe case patients, there is a reduction of peripheral blood T cells and an increase of pro-inflammatory cytokines in the plasma (García, 2020; Yuki et al., 2020; Zhou et al., 2020). This change compromises the epithelial-endothelial barrier, leading to the development of pulmonary edema (Wiersinga et al., 2020). Alongside other complications, patients develop ARDS, followed by organ failure caused by viral sepsis in the final stages of the disease (Wiersinga et al., 2020).

Several studies have shown that a multitude of factors could affect the severity of the disease and the immune response. For example, ACE2 expression was found to be present in higher levels in diabetics and smokers (Brake et al., 2020; Cai et al., 2020; Grundy et al., 2020; Wijnant et al., 2020; Reddy et al., 2021). This finding leads to the belief that comorbidities play a significant role in the disease severity and progression, however, this significance needs to be further investigated.

In the earlier stages of the outbreak, preventative measures were put in place to control and reduce the spread of infection, and several available therapeutic drugs have been repurposed for use in COVID-19 (Pradhan et al., 2020; Tu et al., 2020). These repurposed drugs aim by targeting host pathways and/or viral replication mechanisms (Bchetnia et al., 2020; Tu et al., 2020). One of the leading efforts against combating COVID-19 is the development of vaccines, with the Spike (S) protein being used as a novel target (Krammer, 2020; Poland et al., 2020; Tu et al., 2020). Currently, several vaccines are undergoing clinical trials, and the Pfizer/BioNTech and AstraZeneca vaccines have received regulatory approval (Krammer, 2020; Poland et al., 2020; Administration, U.F.a.D., 2020a; Administration, U.F.a.D., 2020b; European Medicines Agency, 2021).

To develop anti-viral drugs and vaccines, it is crucial to understand the molecular basis and pathology of COVID-19. An increase in research output is required to generate data and results at a faster rate; therefore bioinformatics plays a crucial role in COVID-19 research. There is an abundance of transcriptomic data from studies carried out in COVID-19, however, their use is limited by the confounding factors pertaining to each study. The reanalysis of all these datasets in a unified approach should help in understanding the molecular basis of COVID-19. Bioinformatics uses in-silico tools to carry out research on transcriptomic data without requiring the need to be in the lab. Tools and software platforms such as R coding language and NCBI’s GEO database allow for the reanalysis of such data (Wang et al., 2019). In this study, we aim to use the multiple publicly available transcriptomic datasets retrieved from GEO database to identify consistently differential expressed genes in different tissues and clinical settings. This should allow for the identification of COVID-19 biomarkers expressed in patients and the presence of markers specific to disease severity and condition.

## Materials and Methods

### Generating the Datasets

**FIGURE 1:**
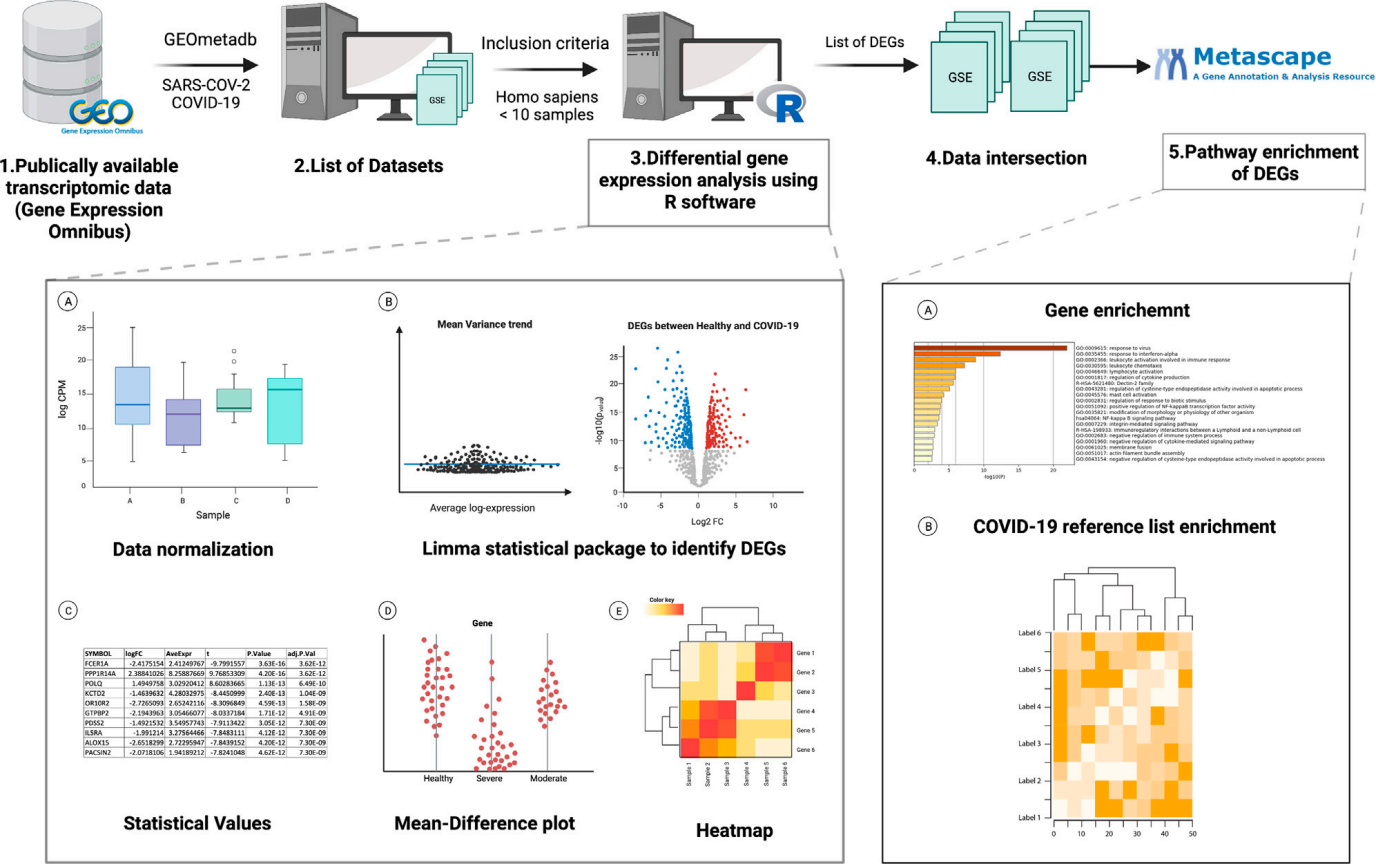
Work flow chart for identifying datasets, R analysis and identifying common differential genes and pathway enrichment analysis (BioRender (2021). Created, 2021).

**FIGURE 2:**
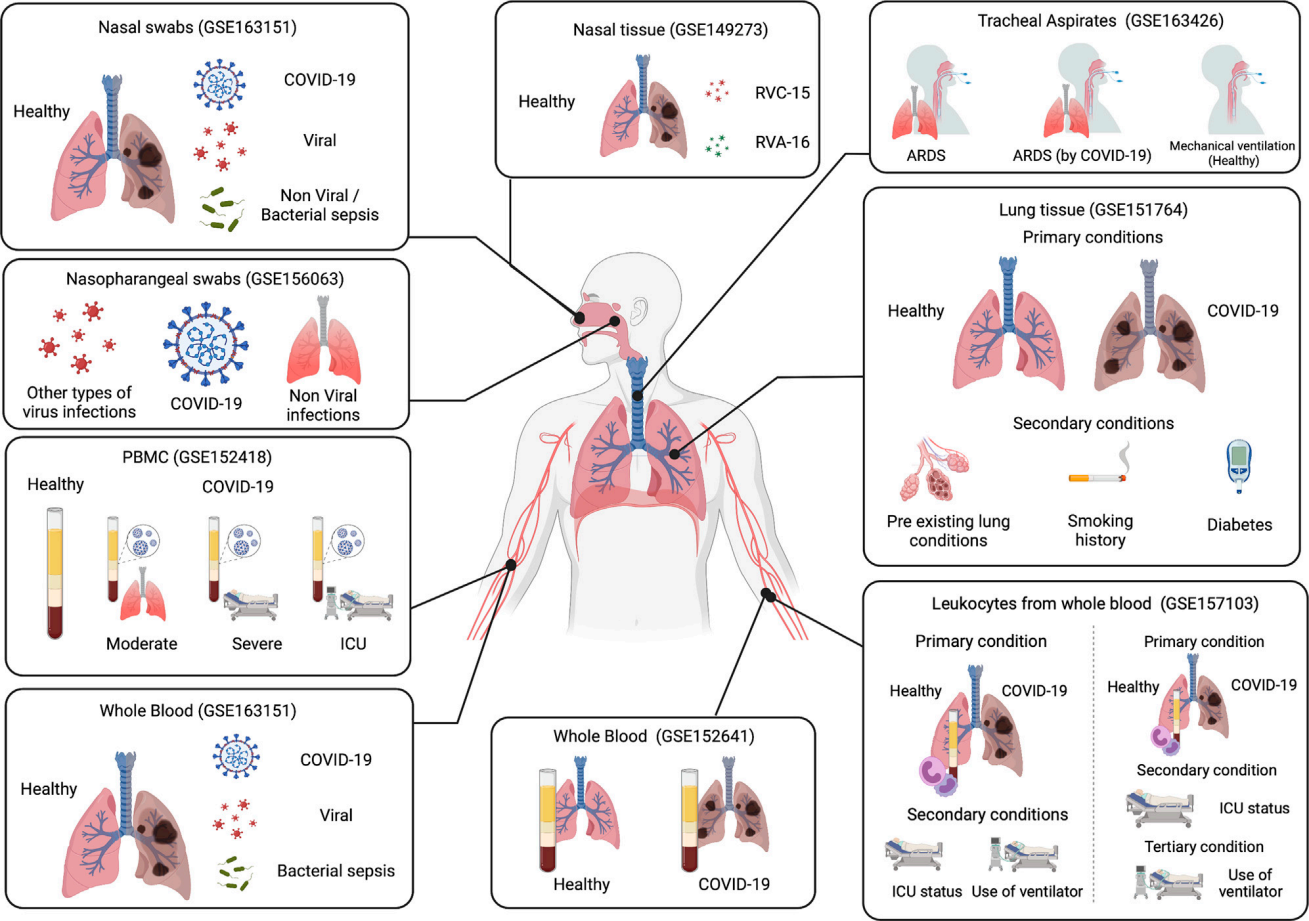
Summary of the different types of analysis carried out (BioRender (2021). Created, 2021).

A list of datasets was generated from NCBI’s GEO database (Barrett et al., 2013) using the GEOmetadb package through the software R (Figure 1). The GEOmetadb package allows the user to query the database efficiently. Query search keywords included SARS-COV-2, and COVID-19. Datasets containing more than 10 samples in human tissues were selected (Table 1). The coding template “RNA seq tutorial” by Lauren Blake was obtained from Rpubs, a R markdown repository, and was used for the basis of this analysis (Blake, 2018). The raw data was imported into R from GEO and the readDGE function was used to create a digital gene expression matrix that was used for further processing. Each dataset was analyzed independently of other datasets (Figure 2). R packages used in this analysis include RNAseq123, gplots, RColorBrewer, R. utils, Limma, Glimma, EdgeR, Homo.sapiens, and were obtained from Bioconductor.

**TABLE 1:** List of Data sets.

GEO accession number	GEO study title	PMID	Source of samples	Number of samples
GSE151764	Two distinct immunopathological profiles in lungs of lethal COVID-19	33033248	Lung tissue	56
GSE163426	COVID-19 ARDS is characterized by a dysregulated host response that differs from cytokine storm and is moderated by dexamethasone	34446707	Tracheal aspirates	52
GSE156063	Upper airway gene expression differentiates COVID-19 from other acute respiratory illnesses and reveals suppression of innate immune responses by SARS-CoV-2	33203890	Clinical naso/pharyngeal swabs	234
GSE149273	RV infections in asthmatics increase ACE2 expression and stimulate cytokine pathways implicated in COVID19	32649217	Nasal Tissue	90
GSE163151	A diagnostic host response biosignature for COVID-19 from RNA profiling of nasal swabs and blood	33536218	Nasal Swabs Whole blood	404
GSE152641	Transcriptomic Similarities and Differences in Host Response between SARS-CoV-2 and Other Viral Infection	33437935	Whole Blood	86
GSE151161	Blocking of the CD80/86 axis as a therapeutic approach to prevent progression to more severe forms of COVID-19	34075090	Whole Blood	79
GSE152418	Systems biological assessment of immunity to severe and mild COVID-19 infections	32788292	PBMC	34
GSE157103	Large-scale Multi-omic Analysis of COVID-19 Severity	33096026	Leukocytes from whole blood	126

### Sample Annotation

Samples were annotated in R using the Homo. sapiens annotation package. The gene identidiers provided with the data was used to match the sample gene ID to the gene name in the annotation database. Samples were grouped based on the disease status and other conditions for analysis.

### Data Filtering and Normalization

Data were filtered to remove all duplicate genes and the log counts per million (CPM) was calculated for genes with at least one count in three different samples. Samples were then normalized using the ‘calcNormFactors’ function provided with the edgeR package; the trimmed mean of M-value (TMM) was used as the defult normalization method. Gene expression was normalized following the construction of a linear model. The “voom” function in the limma package was used to calculate the weights of the genes in order to offset the mean-variance relationship created by the model.

### Sample Visualization

Samples were visualized at multiple stages of the analysis. The functions “plotDensities” and “boxplot” were used to visualize the samples during the filtering and normalization process. Multidimensional scaling (MDS) was used to visualize the level of similarity and the relationship between samples based on their grouping. The “plotMDS” and “plotMD” functions were used for this visualization. The Glimma package was used to generate an interactive plot of the sample scaling and DEGs.

### Differentially Expressed Genes

Differentially expressed genes were identified using the limma package. A contrast matrix containg sample data and information was created. The genes per sample were normalized using the “voom” function. Various functions such as ‘fit’, “eBayes”, and “treat” were used to create a linear model for each gene, and calculate various statistics for each contrast of interest in order to test for DEGs. Statistics calculated include t-statistics, log fold change (logFC), *p*-value, adjusted *p*-value, average expression, coefficients, and standard deviations. The “eBayes” and ‘treat’ functions were used to identify DEGs, that could be used for further experimentations. The list of DEGs was visualized in a table and a Venn diagram (Heberle et al., 2015), with the addition of a mean-difference (MD) and a heatmap per contrast. The “Glimma” package was used to generate an interactive MD plot, along with a gene expression plot. For all statistical values, the adjusted *p*-value ≤ 0.05 and logFC ≥2 was used to signify statistical significance.

### Data Intersection and Gene Enrichment

To identify common DEGs among COVID-19 patients, genes from the same datasets, or similar sets in terms of tissues and conditions were intersected and compared with each other (Figure 2). Since several data sets were analyzed at multiple levels, these dataset layers were compared with each other to identify a list of common genes per condition. Gene pathway enrichment and COVID-19 reference list enrichment was carried out in metascape, a gene annotation and enrichment tool, using the identified common DEGs (Zhou et al., 2019).

## Results

### 32 Genes Are Differentially Expressed in COVID-19 Patients

Lung tissues from deceased patients from the dataset GSE151764 were analyzed in this study (Sobottka et al., 2020). Expression of these genes were analyzed at four different levels (Figure 2). In the first analysis, control samples were compared with COVID-19 samples and five genes were identified to be DE in COVID-19 patients (Table 2). The following analyses added a secondary layer to the previous one; control and COVID-19 samples were divided into further subgroups. The second analysis used the presence or absence of pre-existing lung conditions as the secondary grouping criterion. This subgrouping has identified one DEG in COVID-19 patients without any pre-existing lung conditions, while there are 24 genes in those with pre-existing conditions.

**TABLE 2:** List of genes that are differently expressed in GSE151764.

Condition	List of genes
COVID-19	IFI6, OAS3, OAS1, CRTAM, TNFRSF9
COVID-19 without any pre-existing lung conditions	CCR7
COVID-19 with pre-existing lung conditions	IFIT3, GBP1, CRTAM, OAS1, IFI44L, IFI6, OAS3, IFIT2, CXCL11, MX1, ISG15,
	IFIT1, CXCL10, OAS2, TNFSF18, TNFRSF9, LAG3, HLA-G, MS4A1, CD274, GZMK, NCR3, FCGR2B, IL10
COVID-19 in non-smokers	MS4A1, HLA-DOB, CCR7, CRTAM
COVID-19 in smokers	CRTAM, IFI44L, IFIT3, IFI6, OAS1, OAS3, NCR3, MX1, GBP1, IL10, IFIT2, TNFSF18, ISG15, IFIT1, CXCL11, CXCL10, TNFRSF9, HLA-DPA1
COVID-19 without diabetes	MS4A1, CRTAM
COVID-19 with diabetes	IFI27, CXCL11, HLA-DOB, IFI6, OAS3, ISG15, IFIT2, MX1, OAS1, IFIT3, IFIT1, TNFSF14, CRTAM, IL10, IDO1, GBP1, IFI44L, CXCL10, IL1A, IL23A

The third analysis used smoking history for the secondary grouping criteria. People with no smoking history have 4 DEGs and those with a smoking history expressing 18 DEGs. The fourth analysis compared diabetics with non diabetics as a secondary criterion and has identified two DEGS in non-diabetics and 20 DEGs in diabetic patients.

Table 2 contains a list of differentially expressed genes for COVID-19 patients in each specific condition.

### 8 Genes Are Differentially Expressed in ARDS

This study compares tracheal aspirates (GSE163426) taken from three types of patients, those with COVID-19 caused ARDS, those with non-COVID-19 ARDS, and controls with mechanical ventilation (Sarma et al., 2020). A total of eight genes were differentially expressed, five of which are specific in COVID-19 induced ARDS (Table 3) (Figure 6A).

**TABLE 3:** List of differentially expressed genes in GSE163426.

Condition	List of genes
COVID-19 induced ARDS	HSPA14, GBP5, PATL2, FHOD3, PHF11
Non-COVID-19 induced ARDS	ALPK3
Control (Mechanical ventilation)	MEGF6, H2AW

### Upper Airway Gene Expression Reveal Different Genes Are Expressed for Different Types of Infections

The analysis of GSE156063 compares the gene expression of SARS-CoV-2 infections with other respiratory infections, whether they are due to an acute illnesses or viral infections (Mick et al., 2020). Two genes (GZMB & CCL8) were differentially expressed in all cases, 71 genes were differentially expressed in respiratory infections caused by other viral infections, and 24 genes were expressed in nonviral respiratory illnesses (Table 4); (Figure 7A).

**TABLE 4:** List of differentially expressed genes in GSE156063.

Condition	List of genes
Other non-viral acute respiratory illness	IFI6, IFI44L, IFI44, BST2, EPSTI1, IFIT1, ISG15, USP18, IFIT3, CXCL11, OAS3, SIGLEC1, SERPING1, CXCL10, CMPK2, CXCL9, KLHDC7B, RSAD2, IFITM1, CCDC194, OASL, LAG3, IFIT2, UBD
Viral infections	CCL3, IL1RN, SERPINB9, PLEK, TAGAP, PSTPIP2, CCL4, SOCS1, SLAMF7, GBP2, CD274, SH2B2, PIK3AP1, IL1B, CCRL2, GSTA2, FGR, CCL3L3, IFITM2, ICAM1, HCAR3, P2RY14, GPR84, GBP5, CSF2RB, ANKRD66, APOBEC3A, TNFRSF10C, ACOD1, LILRB2, CD69, CD53, FPR2, CD300E, CCR1, HK3, CCL4L2, CREB5, SLC25A47, LILRA5, PDE4B, H2AC18, CSF3R, LCP2, TNFAIP6, G0S2, C5orf58, CASP5, CCL2, MAP6, FPR1, FCGR3B, IL18RAP, SAMSN1, DYSF, TNFRSF1B, GLT1D1, SEC14L3, IL27, BCL2A1, HRH2, AQP9, OSM, TREM1, CXCR1, CLEC4D, SIGLEC14, HSPA6, SCGB1A1, SIGLEC5, PROK2

### Rotavirus Infections Elicit Responses Similar to That of COVID-19

In this study, three different nasal tissue samples from the dataset GSE149273 were comparable: control samples, RVC-15 and RVA-16 infected tissues (Chang et al., 2020). Of all three sample types, only the comparision of rotavirus against control tissues yielded any DEGs. Comparing both rotavirus’ with each other yielded no DEGs. One hundred sixty-six genes were differentially expressed in control samples one in RVC-15 and 49 in RVA-16 (Supplmentary Figure S2). Table 5 contains the list of DEGs for each condition.

**TABLE 5:** List of differentially expressed genes in GSE149273.

Condition	List of genes
RVC-15	GIMAP2
RVA-16	DHX58, STAT2, NMI, LAP3, SCO2, TOR1B, STARD5, ZNFX1, PSMB9, TRIM69, NUPR1, APOL2, C17orf67, NCOA7, HESX1, TNFRSF6B, PPM1J, BIRC3, STX11, CEACAM1, IL15RA, TLR2, IL19, OR52N4, PDGFRL, GBP2, MUC13, CXCL8, IL7R, PLAUR, RIMS2, BATF3, CXCL3, IL36G, CASP5, OR51B5, PLCL1, FXYD6, PDCD1LG2, CXCL2, CCL3, SLC26A4, LINC01208, PTGS2, TNFAIP6, DEFB4A, DEFB4B, CHRNA1, CCL2
Control	MX2, CMPK2, IRF7, RSAD2, OAS3, DDX60L, MX1, IFIT1, USP18, TRIM25, IFIT5, STAT1, OAS2, OAS1, XAF1, IFIT2, DDX58, EPSTI1, PNPT1, IFIT3, OASL, SAMD9L, HERC5, LAMP3, HERC6, IFI35, RTP4, PARP12, PARP9, ISG15, UBE2L6, HSH2D, C19orf66, IFI44, TRANK1, IFIH1, SP110, PLSCR1, IFITM1, DDX60, PARP10, PARP14, TRIM21, ISG20, ETV7, NLRC5, SLC25A28, IFI44L, TRIM22, RNF213, SAMD9, NT5C3A, PML, GBP4, TAP1, TNFSF10, PLEKHA4, LMO2, GBP1, APOL6, EIF2AK2, HELZ2, CXCL10, BST2, TYMP, GMPR, IFITM3, BATF2, SECTM1, IFI6, APOBEC3G, WARS, AIM2, EXOC3L1, BCL2L14, LIFR, GBP5, SLC15A3, ZBP1, FBXO39, HSPB9, PIK3AP1, RASGRP3, MYH7, MLKL, APOBEC3A, GBP1P1, DUOX2, RUFY4, DUOXA2, FGD2, USP30-AS1, TNFSF13B, CCL5, HRASLS2, GRIP2, IFI27, RBM11, MAB21L2, CD34, HTR2B, SUSD3, SOCS1, CARD17, IL4I1, CD38, TAGAP, LRRN2, CD83, LAG3, SMTNL1, CXCL11, IDO1, CSAG3, CD7, TMEM171, 04-Sep, PLVAP, CSF3, KLHDC7B, NCF1, PTPRR, CD274, CCL20, IFNB1, CXorf49, CXorf49B, NCF1B, XXYLT1-AS2, TNIP3, LINC00890, OR52K2, ART3, RORB, SP140, THEMIS2, PAX5, CCL4, PCDH17, FRMD3, IL17C, LGALS17A, NCF1C, CD69, ZFPM2, TNF, ANGPTL1, IFNL3, EXOC3L4, IFNL1, CLCA3P, FGF2, CYP21A2, SLCO5A1, CACNA1I, SERPINB9P1, CXCL9, IFNL2, ATP10A, IL36A, BCL2A1, NOS2, C3AR1, IL6, TRIM31, WISP1

### Nasopharyngeal Swabs Are a Better Diagnostic Tool for Sequencing COVID-19 Compared to Whole Blood

In this study, samples were taken by both nasopharyngeal swab and whole blood (GSE163151) (Ng et al., 2020). Each sample source was analyzed independently from the other and 119 genes were found to be DE in COVID-19 patients from nasopharyngeal swab samples, while in whole blood samples, 10532 genes were identified (Figure 10).

In the nasopharyngeal swab samples, 467 genes were DE in control samples, 1865 in non-viral/bacterial cuased acute respiratory illness samples, and over 4,000 genes were differentially expressed in viral infections. On the other hand, the whole blood samples have 187 DEGs in controls, 2,262 in bacterial sepsis (non-viral) acute respiratory illness samples, and 193 genes in viral samples.

### There Are 79 Genes Differentially Expressed in COVID-19 Patients From Whole Blood Samples, Most of Which Are Upregulated

Whole blood samples from healthy controls and COVID-19 patients were used (GSE152641) (Thair et al., 2020). The analysis showed that 79 genes are differentially expressed between conditions, of which 61 genes are upregulated in COVID-19 infections, and 18 are down-regulated.

### Abatacept Treatment Yielded No Differential Expression of Genes Compared to Control Samples

Taken from the dataset GSE151161, this study tested the use of abatacept in rheumatoid patients with a pathology similar to that of COVID-19 patients (Julià et al., 2020). Whole blood samples were sequenced before treatment (Week 0) and after treatment (Week 12). The analysis revealed no genes that are differentially expressed between control and treatment samples.

### Severe COVID-19 Patients Share all Differential Expressed Genes With That of ICU Patients

The samples from this study are collected from peripheral blood mononuclear cells (PMBC) (GSE152418) and analyses the differences between COVID-19 disease severities (Arunachalam et al., 2020). The first comparison analyzed control samples with COVID-19 samples, resulting in 371 differentially expressed genes. Then, a second analysis was carried out based on the severity level to identify which DEGs belong to what conditions. Five hundred and four genes were identified to be differentially expressed in healthy patients, four genes in moderate patients, four in severe, and 156 in ICU patients (Table 6).

**TABLE 6:** List of genes that are differentially expressed in GSE152418.

Condition	List of genes
Moderate	OTOF, TNFRSF10C, HK3, AXL
Severe	SAMD14, CMTM5, PLXNB3, MYL9
ICU	TGFB1I1, CMTM5, MYL9, SAMD14, CETP, TTC7B, NT5M, MSRB3, EGFL7, ARHGAP6, MMRN1, SPX, LNCAROD, CDC42BPA, XK, TREML1, SMOX, PLOD2, GSTM5, PLXNB3, MFSD2B, CAV2, CLU, ITGA2B, SCN1B, TTLL7, PROS1, SELP, PF4V1, PCSK6, ITGB5, NRGN, ABLIM3, SPTB, FAM20A, CALD1, MPIG6B, PCOLCE2, EGF, WFDC1, PTGER3, GP9, AC015912.3, MGLL, LCN2, PF4, AQP10, LY6G6F, TFPI, FSTL1, ELOVL7, ANKRD9, PEAR1, CTTN, BEX3, GP6, VWF, ANXA3, SPARC, LY6G6E, MAOB, LINC01151, ACRBP, CLEC1B, GP1BA, PPBP, ALOX12, LTBP1, PRKAR2B, ITGB3, PTPRF, CTDSPL, FAXDC2, MYLK, WASF3, VEGFC, MAP1A, EHD3, TNNC2, GAS2L1, PTGS1, MMP8, GNAZ, KREMEN1, AL162424.1, ARHGEF12, ITGA1, PCYT1B, MED12L, VSIG2, CLDN5, PGAM1P8, RETN, AL450468.2, ESAM, METTL7B, CREB3L1, HLX, TRIM7, JAM3, PRTFDC1, WASF1, MAFB, SH3BGRL2, ITGA7, F2RL3, VEPH1, WDFY3-AS2, TAL1, F13A1, AC090409.1, S100A8, SAPCD2, KAZN, GUCY1B1, LINC01503, SLC25A37, TLCD4, FAM20C, S100A9, S100A12, GJA4, HTRA1, KRT80, LHFPL6, LYVE1, TREML3P, AC215522.2, GRB10, PLPP3, SLC18A2, ASGR2, VSIG4, MGST1, TRAJ32, AC092490.1, ADAM9, STAB1, MCEMP1, THBS1, DYSF, ZNF185, NIBAN2, FLVCR2, PLBD1, AQP9, FPR2, PTAFR, DLC1, VNN3, CYP1B1-AS1, VNN1, ACSL1, SIRPA, SERINC2, CYP1B1

### Genes Linked to Cell Division Are Differentially Expressed in COVID-19 Infections

With over 100 samples obtained from leukocytes (GSE157103), this is one of the larger studies that were reanalyzed (Overmyer et al., 2020). This study was analyzed at four levels (Figure 2), similarly to GSE151764 reanalysis. The first analysis compared the gene expression between control samples and COVID-19 samples. Following that, a secondary division was carried out, with an analysis comparing ICU patients with non-ICU patients, and another analysis comparing ventilation use with patients not requiring ventilation. The fourth analysis used a tertiary division; therefore, it compares samples by disease state, followed by ICU status, and finally ventilation requirement.

The first analysis identified 115 genes to be differentially expressed. The added levels of comparisons gave more perspective to the DEGs distribution, with the following number of genes identified in each analysis (Table 7).

**TABLE 7:** Number of differentially expressed genes in each analysis.

First level: Control vs COVID-19	First level: Control vs COVID-19	First level: Control vs COVID-19
**Second level:** ICU vs Non-ICU	**Second level:** Ventilation vs non ventilation	**Second level:** ICU vs Non-ICU
**—**	**—**	**Third level:** Ventilation vs non ventilation
Control ICU: 2 genes	Control Ventilation: 12 genes	Control non-ICU and non-ventilation: 491 genes
Control Non ICU: 308 genes	Control non-Ventilation: 179 genes	COVID-19 non-ICU and ventilation: 2 genes
COVID-19 ICU: 431 genes	COVID-19 Ventilation: 394 genes	COVID-19 non-ICU and non-ventilation: 11 genes
COVID-19 non-ICU: 34 genes	COVID-19 non-Ventilation: 13 genes	COVID-19 ICU and ventilation: 377 genes
—	—	COVID-19 ICU and non-ventilation: 1 gene

## Discussion

### CRTAM Is Found to Be a Common Gene Among COVID-19 Patients With Varying Comorbidities

Following the gene expression in COVID-19 severity, it is important to look at secondary conditions and comorbidities when assessing disease progression and which genes are associated with what.

**FIGURE 3:**
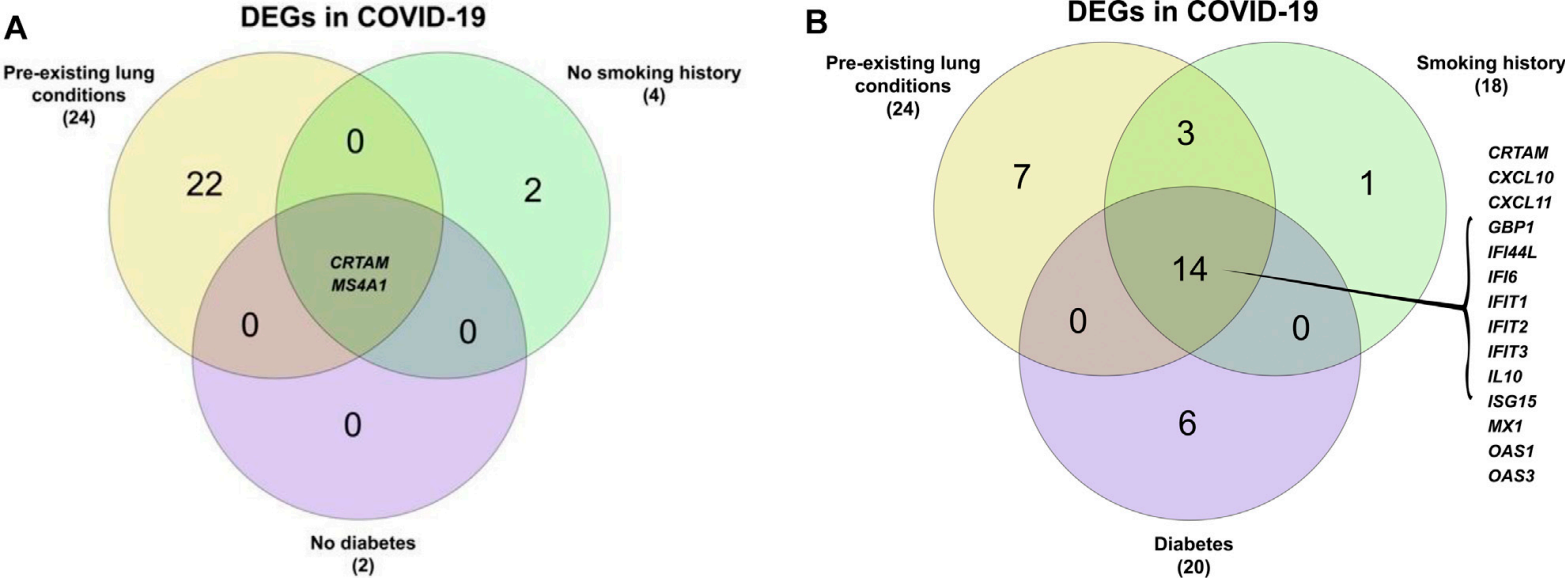
Intersecting the differential expressed genes of **(A)** COVID-19 with pre-existing conditions, no smoking history, and no diabetes. **(B)** COVID-19 with pre-existing conditions, diabetes, and a smoking history.

**FIGURE 4:**
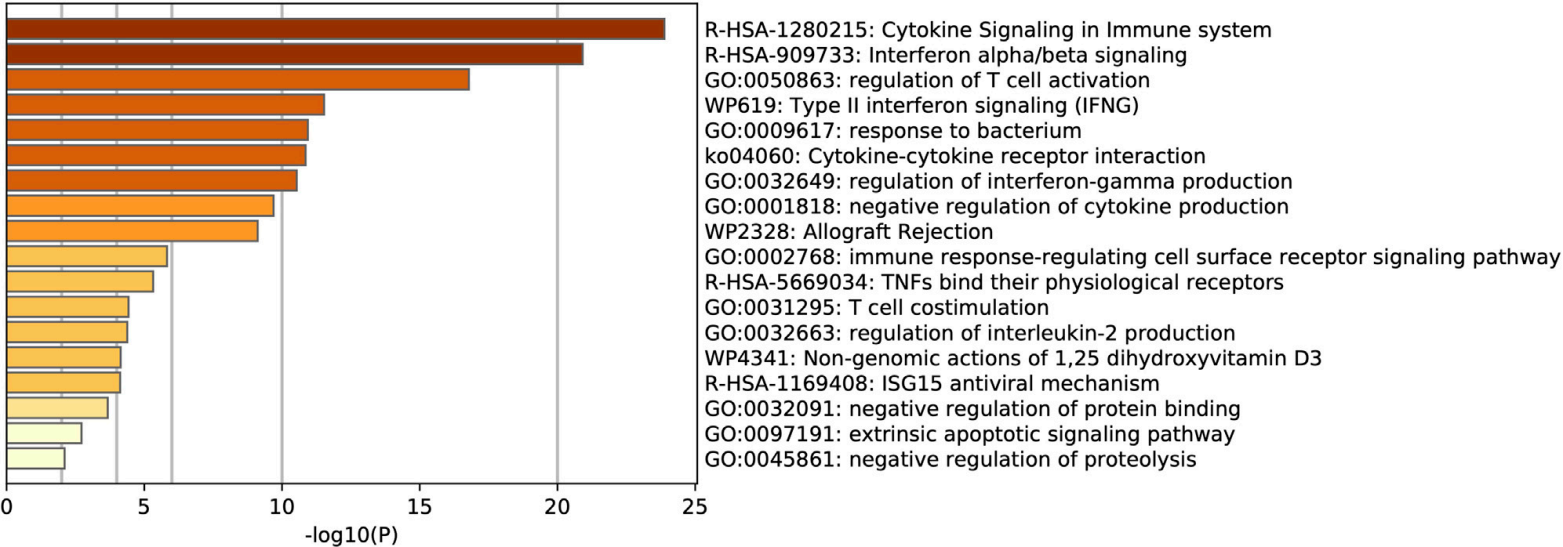
Gene term enrichment analysis of the DEGs for COVID-19 in GSE151764 reveals that most genes are involved in the cytokine signaling of the immune system.

Due to this study being analyzed at multiple levels, it was possible to intersect and compare the data. CRTAM was identified as a common gene among COVID-19 patients, regardless of their smoking history and diabetes status (Figure 3). CRTAM, which stands for cytotoxic and regulatory T cell molecule, is the gene responsible for regulating the activation and differentiation of several T-cell subsets such as NK cells. This could indicate that the gene is essential in the prognosis of COVID-19, as it is down-regulated in COVID-19 patients and more so in those with pre-existing lung conditions, smokers and diabetics. Additionally, 14 genes are differentially expressed in SARS-CoV-2 patients, including MX1, OAS1, and OAS3, all of which are involved in anti-viral responses, including cytokine signaling (Figure 4).

There are three genes shared between SARS-CoV-2 patients that have pre-existing lung conditions and smoking history, which are TNFSF18, TNFRSF9, NCR3. These genes are involved in cytokine signaling and regulation of T cell activation (Figure 4). No genes are shared between pre-existing lung conditions and diabetes, while diabetics have 6 DEGs in that indication. These common genes differ based on the sets compared, hinting that certain genes are affected by various factors, some more so than others.

**FIGURE 5:**
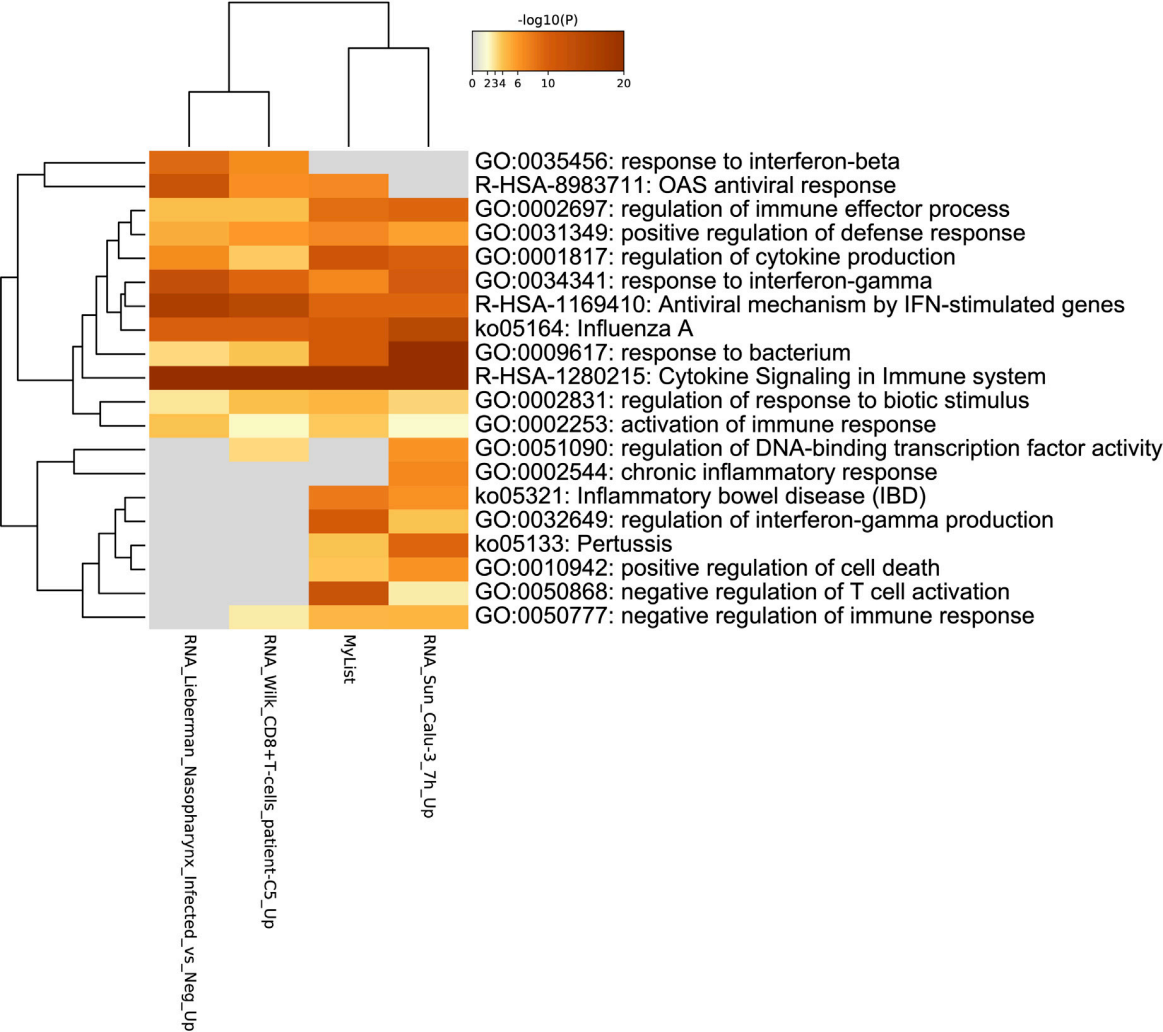
Gene term enrichment against COVID-19 reference list for the DEGs in COVID-19 reveal that the reactome gene sets for cytokine signaling are involved in COVID-19 prognosis.

Enrichment analysis of COVID-19 pathways (Figure 5), reveals that the majority of the genes are involved in the cytokine signaling pathways, followed by regulation of cytokine production and response to bacterium pathways.

### Genes Differentially Expressed in COVID-19 Induced ARDS Are Involved in Protein Complex Assembly

**FIGURE 6:**
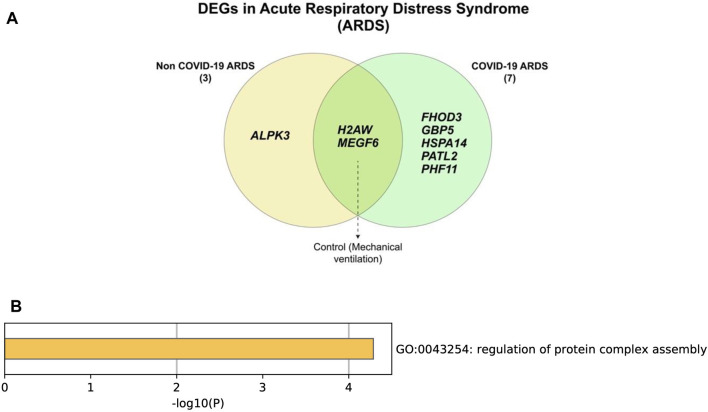
**(A)** Differentially expressed genes in ARDS caused by COVID-19. And **(B)** enrichment of COVID-19 induced ARDS genes reveal that most genes are linked to protein regulation.

Of the five DEGs in COVID-19 caused ARDS, three genes (PATL2, FHOD3, and HSPA14) were found to be linked to protein complex assembly when enriched, either through nucleotide biding or by protein binding (Figure 6). On the other hand, two genes are not involved in regulating protein complex assembly; those are GBP5, which has a role in the innate immune system and inflammation, and PHF11, which is linked to asthma. Regardless, the other three ARDS-causing genes could be attributed as part of the body mechanisms instead of the COVID-19 viral-induced mechanisms. When attempting to enrich the COVID-19 pathways, none of the five COVID-19 ARDS-causing genes were found to be linked to any of the pathways (Supplmentary Figure S1).

### Differentially Expressed Genes Are Involved in Cytokine Signaling and Antiviral Mechanisms

**FIGURE 7:**
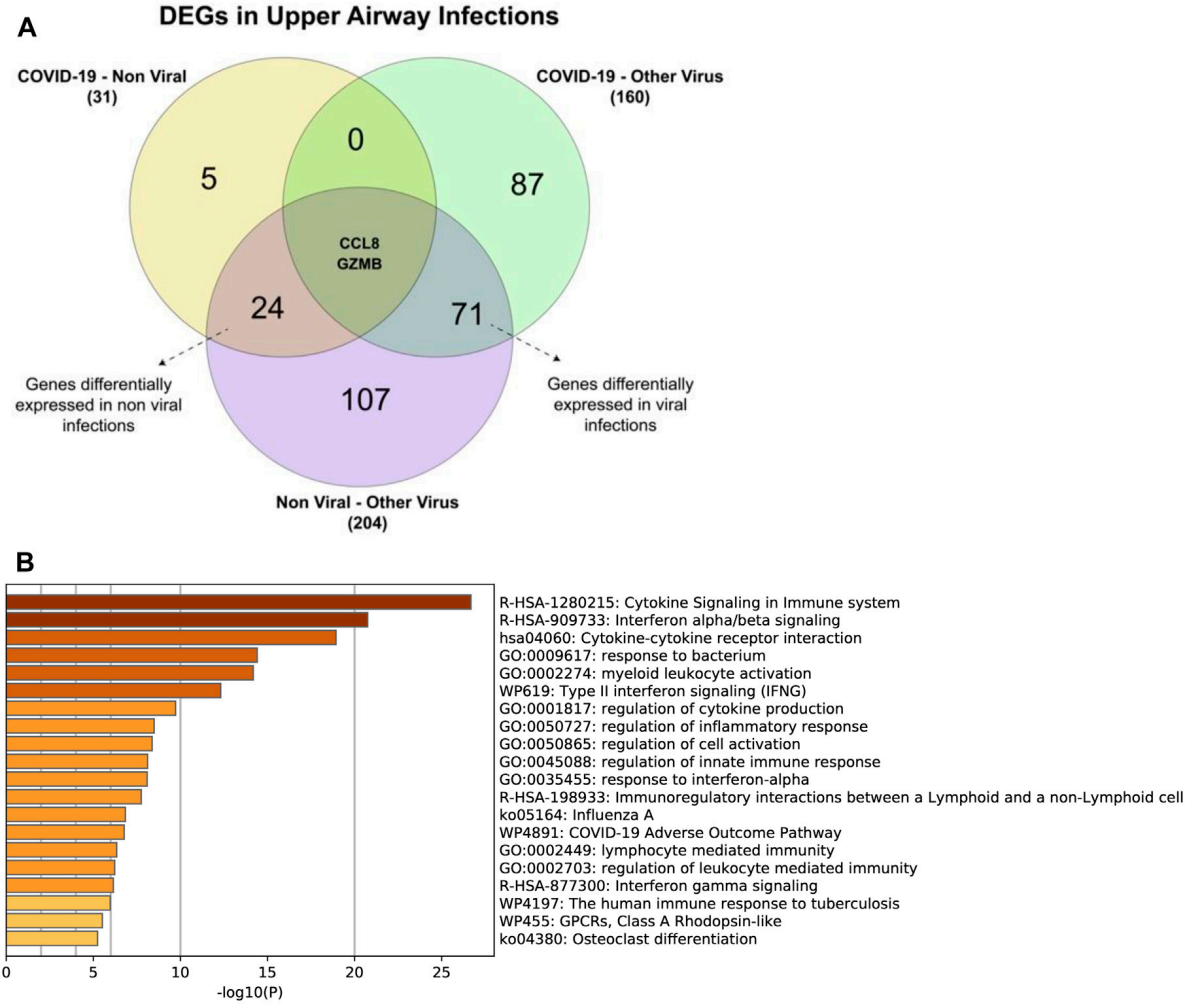
**(A)** Number of genes differentially expressed genes between the different types of upper airway infections. And the enriched terms for genes that are differentially expressed in **(B)** all types of respiratory diseases.

**FIGURE 8:**
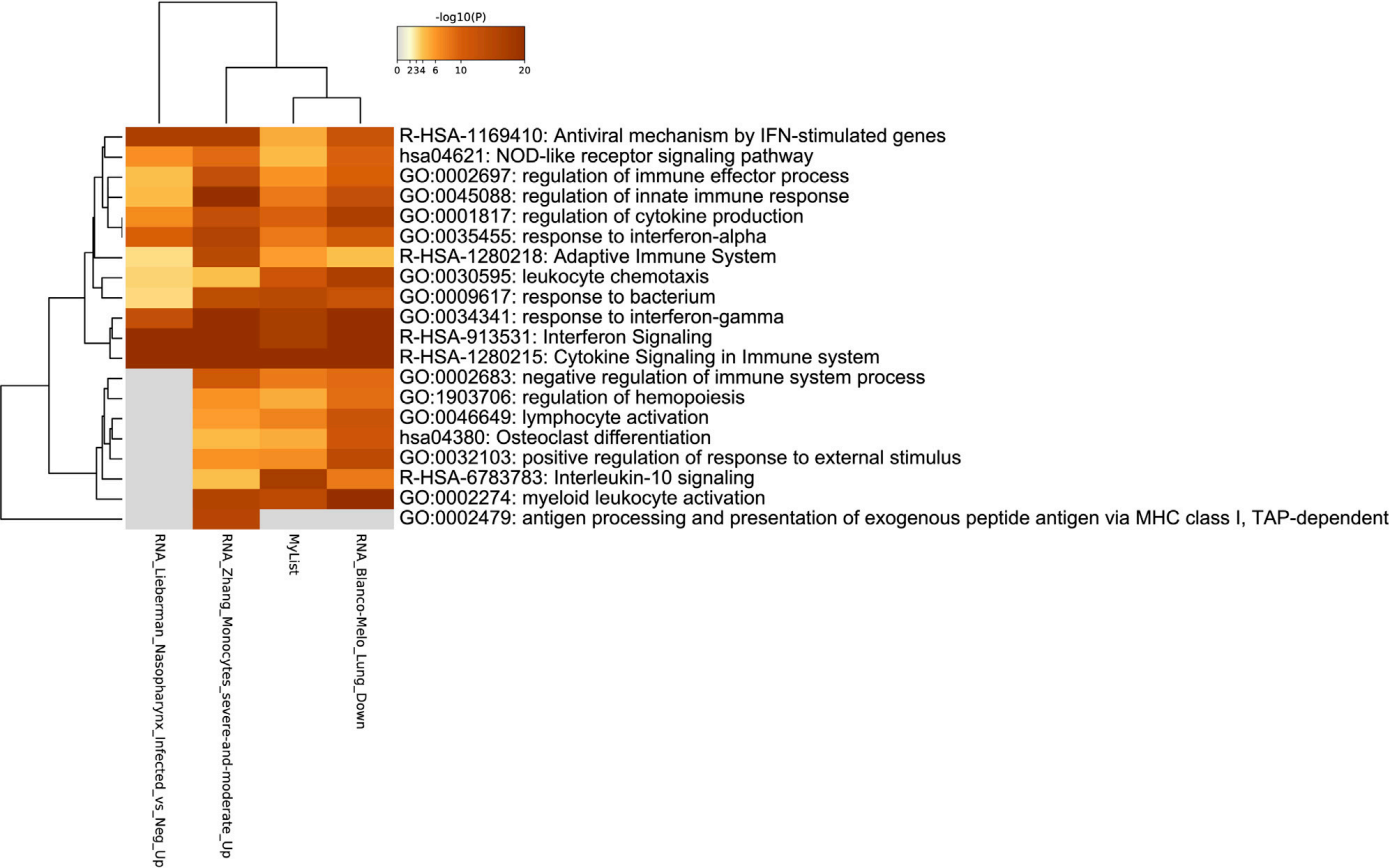
Heatmap of enriched genes for COVID-19 related pathways in upper airway infections.

While identifying DEGs of COVID-19 is important, the disease is caused by a virus, and all viruses elicit similier immune responses. Therefore, comparing the differential expression of COVID-19 to that of other respiratory illnesses caused by both viral and nonviral factors is cruical. Two DEGS were identified in all types of infections (Figure 7A). Gene enrichment of all 97 common genes shows that most of the identified genes are involved in cytokine signaling and the immune responses (Figure 7B). Enrichment against COVID-19 reference lists (Figure 8) reveals that most of these genes are specific for cytokine signaling; this is because across the gene input list, only two genes were found to be common between SARS-CoV-2 and other illnesses while the others are differentially expressed for other respiratory illnesses. This serves as an indicator that the potential list of genes that could be used as COVID-19 specific biomarkers is small.

### Rotavirus Infections Induce Cytokine Signaling Pathways and upregulate MX1, MX2, and Various Interleukins

Our reanalysis validated this study’s findings as ACE2 expression has been upregulated in both RVC-15 and RVA-16 samples. Additionally, MX1 and MX2, genes that are associated with anti-viral response, were upregulated in the viral infected samples. Furthermore, various interleukins have also been upregulated in the rotavirus infected samples, including IL7R, IL19, and IL4I1. The implication of ACE2 being upregulated in other viral infections reveals that COVID-19 is not the only virus that upregulates this gene. This further proves the need to compare the identified DEGs to that of other viruses and/or illneses, especially of those with similar structure or pathology.

**FIGURE 9:**
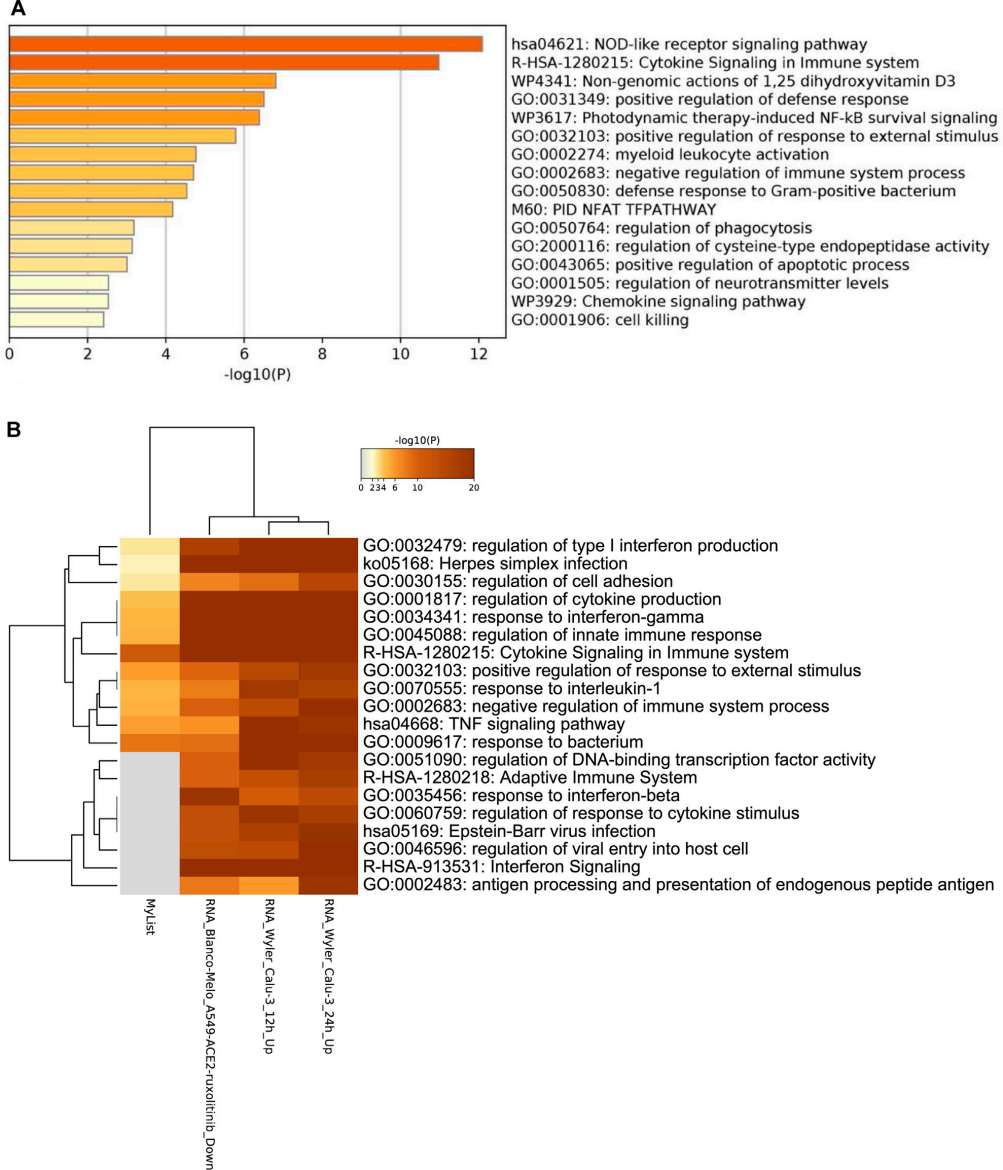
**(A)** Gene term enrichment for the differential expression genes in RVA-16 and RVC-15 **(B)** Gene term enrichment for COVID-19 pathways.

Figure 9A reveals the enriched pathways of the 51 DEGs in both RVA-16 and RVC-15. The two most expressed pathways are that of NOD-like receptor signaling pathway and the cytokine signaling in the immune system. Both pathways are part of the immune system’s response to pathogens. Most of the pathways involve the immune system response to pathogenic invasion, such as activating myeloid leukocytes, resulting in an immune response that affects other pathways. Of the RVA-16 and RVC-15 differential expressed genes (Figure 9B), not many are shared with COVID-19 gene lists, and if shared they are at low levels. However, the cytokine signaling in the immune system pathway has the highest level of enrichment, indicating that rotavirus’ can stimulate the same cytokine pathways involved in COVID-19 infections.

### Genes That Are Common in Both Nasopharyngeal Swabs and Whole Blood Sequencing Are Linked to Interferon Signaling and Response to Interferon-Alpha

**FIGURE 10:**
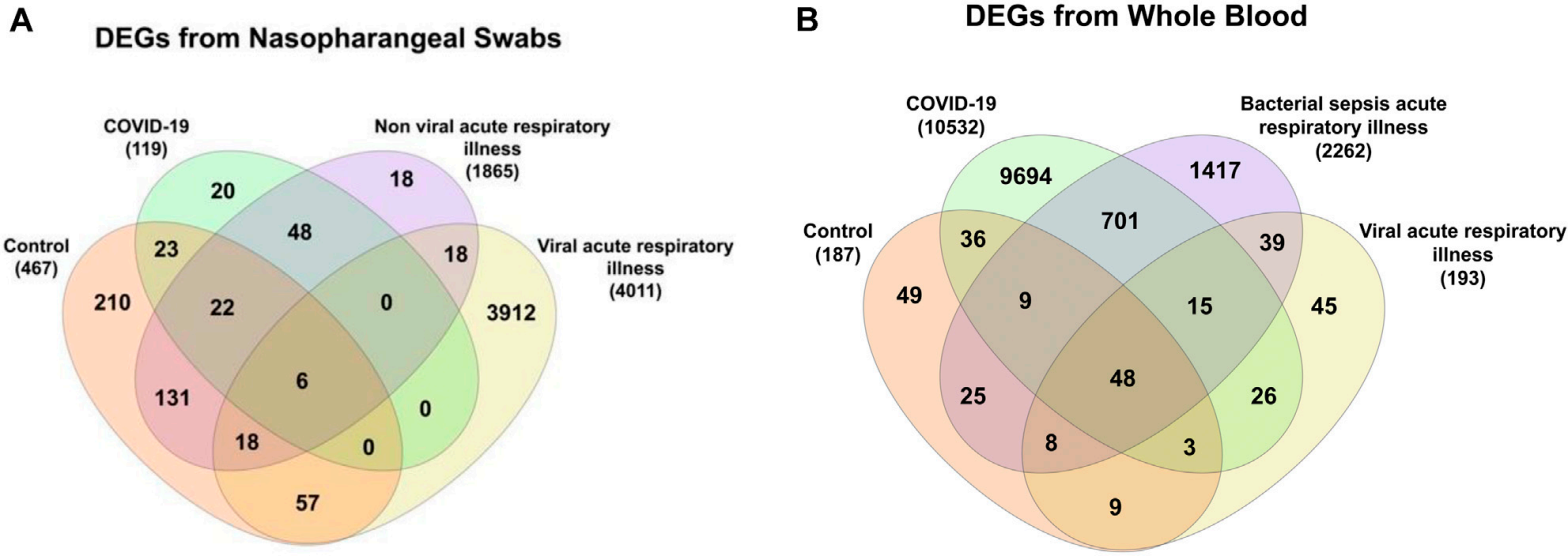
Common DEGs between controls, COVID-19 patients, and patients from viral or nonviral acute respiratory illness from **(A)** nasopharyngeal swabs and **(B)** whole blood samples.

Nasopharyngeal swab samples, reveal 48 genes that are common between COVID-19 and nonviral acute respiratory infections (Figure 10A). Only 20 DEGS are specific to COVID-19 that are not shared with any other indications (Table 8), and therefore, could be used as potential biomarkers.

Whole blood samples had a higher number of DEGs in COVID-19, including DEGs specific to COVID-19 (Figure 10B). Of these genes, 701 were common with bacterial sepsis, and 26 were common with other viral infections.

**FIGURE 11:**
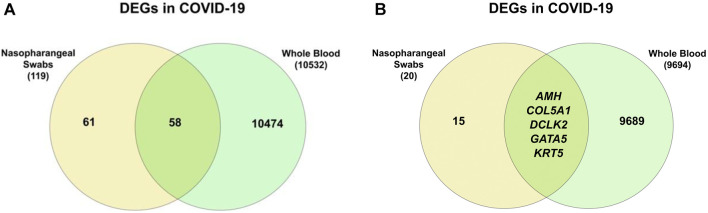
**(A)** From the COVID-19 DEGs, 58 genes are common between nasopharyngeal swabs and whole blood samples **(B)** five genes are common from the COVID-19 specific DEGs.

This difference in the number of differentially expressed genes is expected due to the different tissue sources used for sequencing. When comparing the COVID-19 DEGs from both sample sources, 58 genes are found to be common (Figure 11A). However, comparing the COVID-19 specific DEGs, five genes were found to be common between the two sample types (Figure 11B).

**FIGURE 12:**
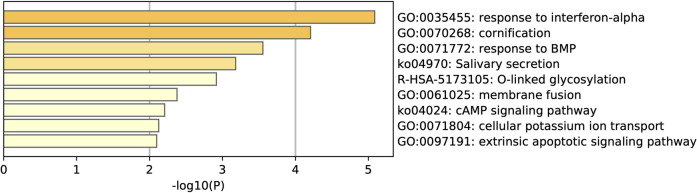
Pathway enrichment for the 58 genes that are common in COVID-19 patients between nasopharyngeal swabs and whole blood samples.

Gene enrichment through metascape reveals that response to interferon-alpha is the most enriched pathway (Figure 12). Other enriched pathways include GO:0070268 cornification, which is linked to cell death, and GO:0071772 response to BMP, which results from a response of growth factors. Enriching against COVID-19 reference lists, reveals that the interferon pathway is the only common pathway between this list of DEGs and the publically available list of COVID-19 genes (Supplmentary Figure S3). This finding helps in understanding the clinical differences between responses present in the site of infection (nasal/pharynx) compared to that of the immune system (blood circulation) and how sample locations affects sequencing results.

### Differentially Expressed Genes Are Linked to Viral Entry and the Immune Response

**FIGURE 13:**
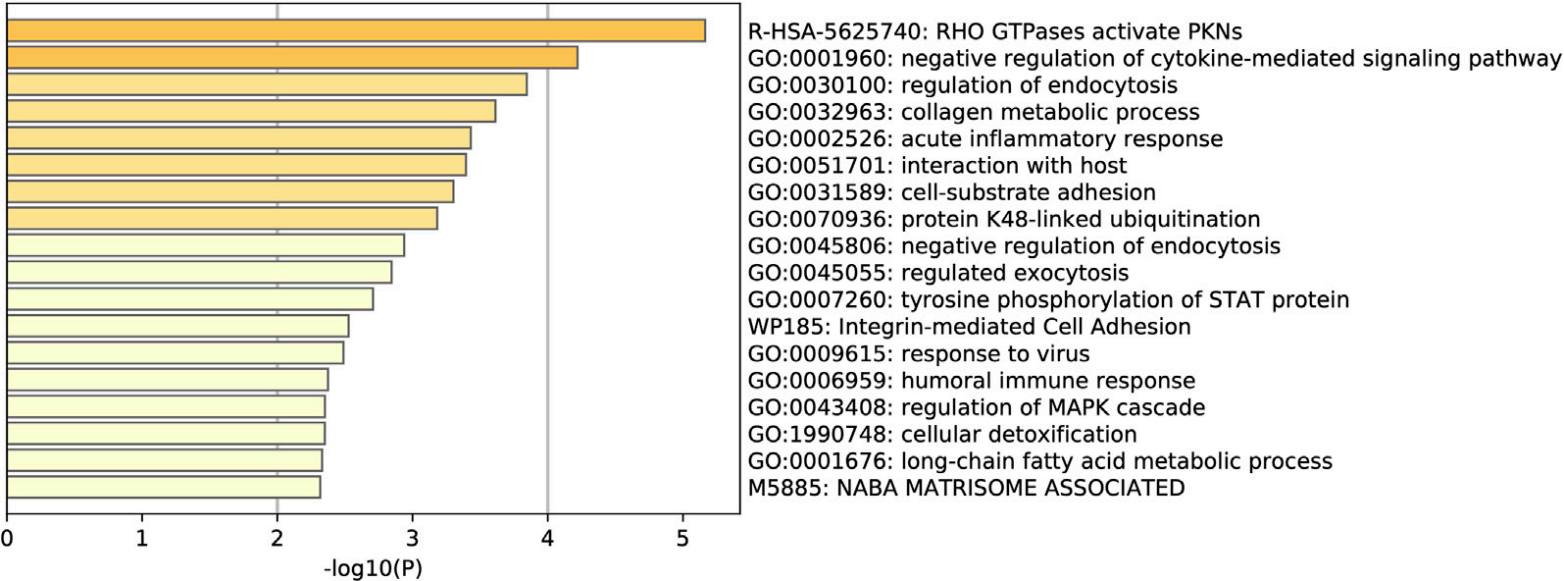
List of gene terms enrichment for the upregulated COVID-19 genes in GSE152641.

Off the 79 DEGs in GSE152641, the ratio of upregulated genes to downregulated genes in COVID-19 was higher (Table 9). Some of the upregulated genes include SIGRR, NOG, SDC1 and IGFBP2, all of which are involved in the negative regulation of cytokine signaling (Figure 13). Other genes are involved with various immune pathways and anti-viral responses, such as those genes involved in interaction with the host. Though in comparision with COVID-19 reference lists, enrichment for the DEGs is low, with the highest level of enrichment is that for autoimmune disease systemic lupus erythematous, followed by leukocyte migration and viral entry into the host cell (Supplmentary Figure S4).

**TABLE 8:** List of genes that are differentially expressed in COVID-19 analyzed from GSE163151.

COVID-19 (nasopharyngeal swabs)	IFI6, IFI44, IFIT3, IFIT2, AMH, EIF2AK2, GBP1, IFI27, KRT5, GBP5, CXCL10, HS1BP3, ADCK2, ZBP1, SNORA61, HSF2, RNVU1_8, COL5A1, GATA5, DCLK2
COVID-19 (common between nasopharangeal swabs and whole blood)	AMH, KRT5, COL5A1, GATA5, DCLK2

The genes involved in the viral entry are of importance due to their link with the virus, as preventing viral access to the host is one of the main courses of action for preventing COVID-19. However, a point of importance is that while these pathways are enriched, they are done at low levels compared to the other COVID-19 lists.

### Transcriptomic In-Silico Studies Are Not Enough to Validate Data

Our results for this analysis have shown no differential expression of genes; however, the study (GSE151161) has carried out further experiments to validate their results. Therfore, this analysis is a reminder to not depend entirely on data and results generated in-silico and that further validation is required. We should use more than transcriptomics to answer our questions.

### Hemostasis Is the Most Enriched Pathway for COVID-19 Patients

Disease progression is dependent on the immune response to viral infection and the genetic predisposition a patient has. When intersecting and comparing the differentially expressed genes for each severity, all DEGs for severe patients were common with those of ICU patients (Supplmentary Figure S5). It is important to note that gene expression levels between severe and ICU are similar to each other due most severe patients ending up in the ICU. The genes commonly expressed in severe and ICU patients are associated with GO processes involving the regulation of cell differentiation. Furthermore, 70 DEGs are shared between healthy and severe COVID-19 patients, while all four differentially expressed genes for moderate disease state is specific for those with moderate disease. These common genes between healthy and ICU patients are noteworthy due to their presence in these two conditions. One of these genes is HLX, which is found to be upregulated in healthy patients compared to COVID-19 patients. However, when comparing HLX’s expression based on severity levels, this gene is upregulated in ICU patients in comparision to the other disease states and healthy individuals. This difference in expression based on the state of severity indicates that there are potential genes that are linked to the disease state rather than the disease as a whole.

**FIGURE 14:**
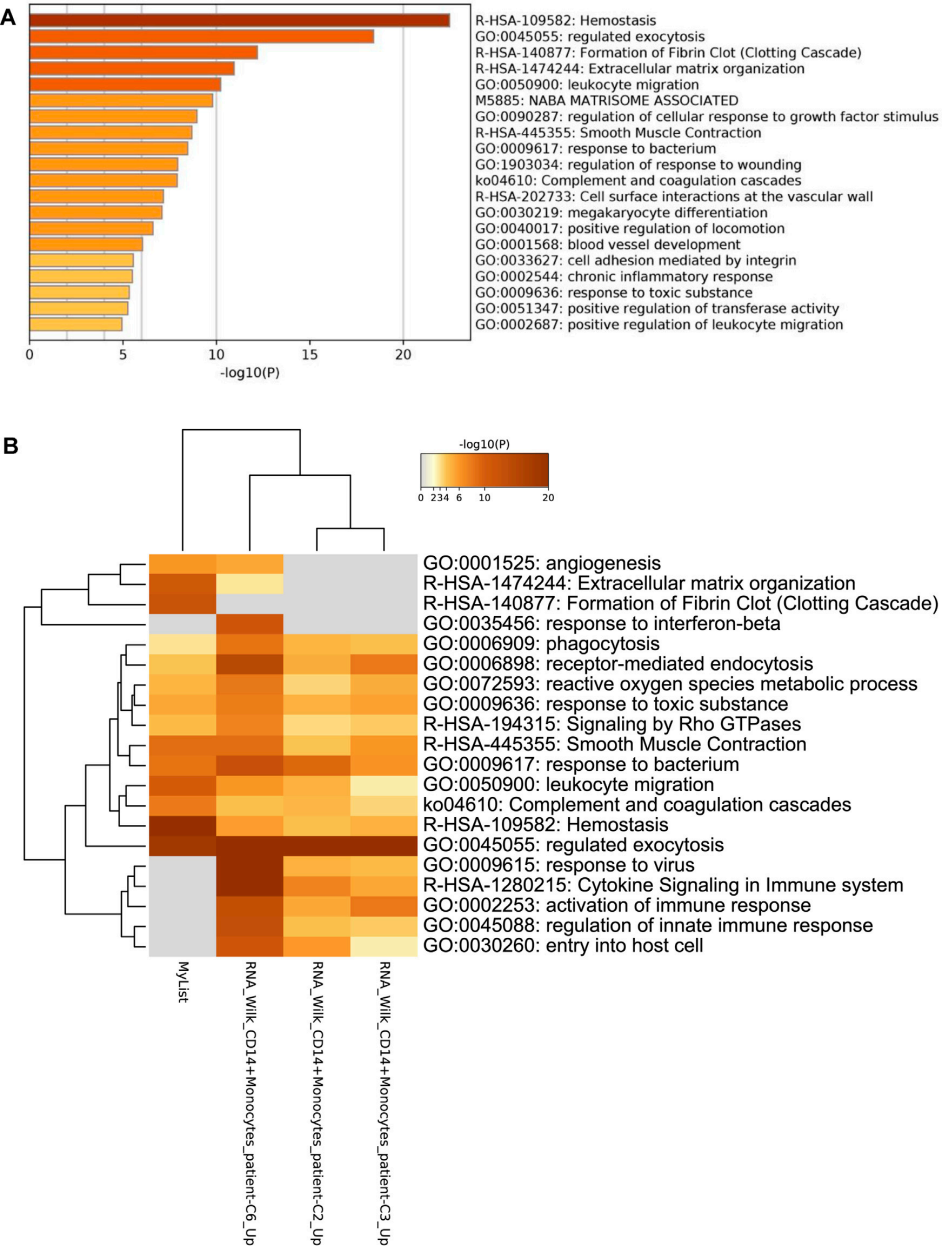
Gene enrichment analysis of **(A)** regular pathways is showing that the most common pathway is that of hemostasis and exocytosis and are all linked to cell differentiation. While in **(B)** COVID-19 gene lists, they are followed with angiogenesis, phagocytosis, and several immune responses.

Gene enrichment analysis revealed that in pawthway gene enrichment (Figure 14A), most DEGs are commonly found in the hemostasis pathways, followed by regulating exocytosis. Several of the other pathways are involved in cell differentiation, such as GO:0090287 process, which regulates cell response to growth factors, or R-HSA-1474244 extracellular matrix organization. Following other enriched pathways, all pathways are linked and result in the formation of blood vessels in a chain reaction.

Similarly, when testing against the COVID-19 lists (Figure 14B), similar pathways are enriched, such as the hemostasis pathway, which appears to be highly expressed in this list compared to the other COVID-19 lists. The regulated exocytosis pathway is one of the few pathways that are expressed at similar rates to that of other lists. Unlike the other lists which involve cytokine signaling, this dataset, relies on the complement and coagulation cascades in their immune response.

**FIGURE 15:**
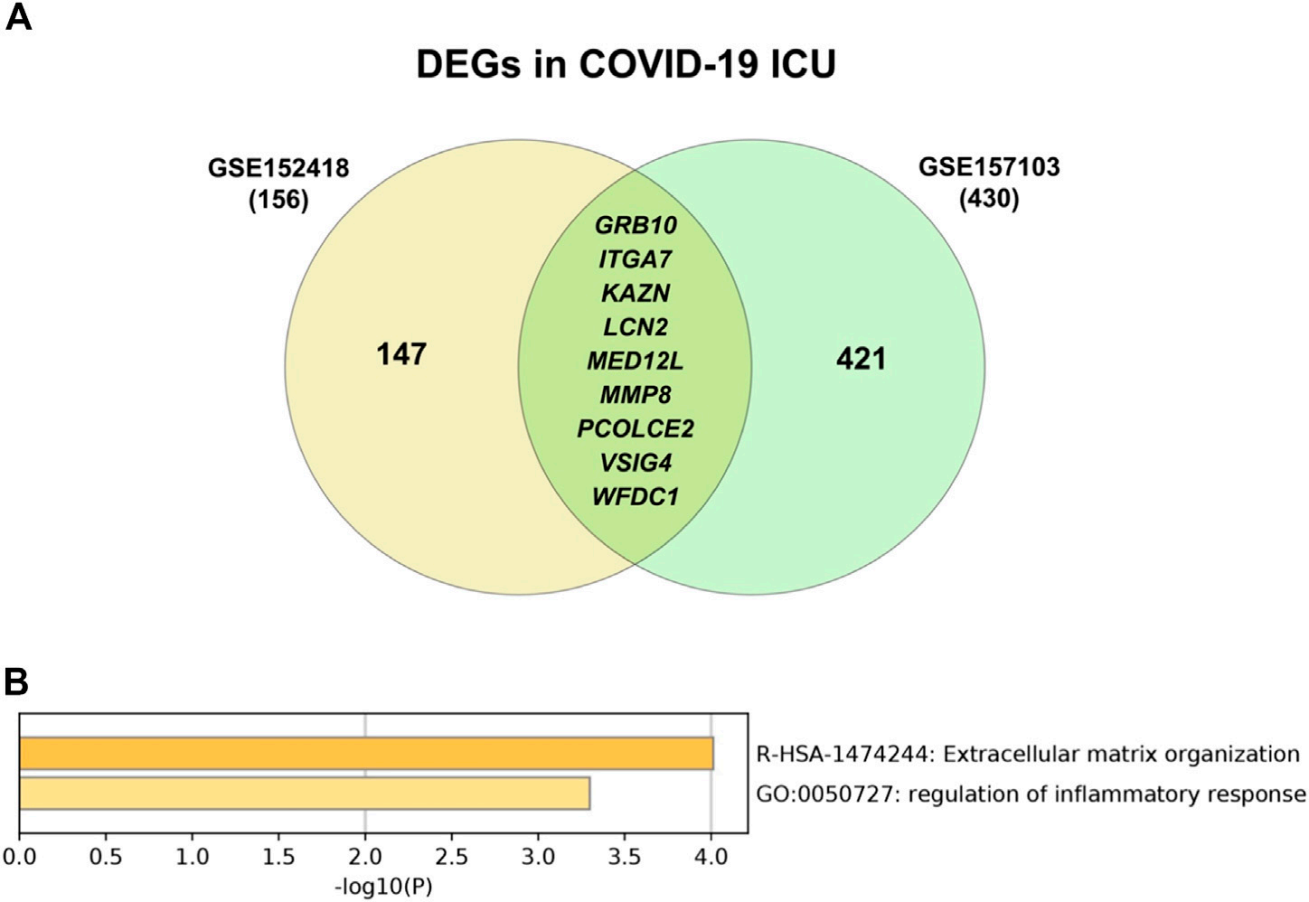
**(A)** Intersecting differentially expressed genes in ICU patients from two different studies (GSE152418 & GSE157103) showed that there are nine genes commonly differentiated in ICU patients **(B)** and these genes are involved in ECM organization and immune response regulation when upon enrichment.

Comparing results of ICU patients from this study with that of another study (GSE157103) there were 9 DEGs common between ICU COVID-19 patients (Figure 15A). Following gene enrichment, these DEGs are involved in extracellular matrix organization and regulation of the inflammatory response (Figure 15B). However, when enriching them to that of COVID-19 lists [Supplmentary Figure S6], only the ECM organization pathway is common among the different lists.

### ICU Patients, Regardless of Ventilation Use, Differentially Expressed Genes That Are Involved With Cell Division

**FIGURE 16:**
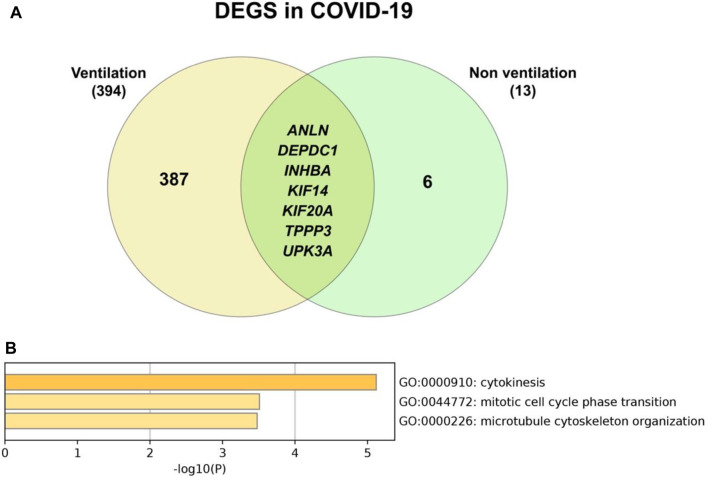
**(A)** Number of genes that are common between COVID-19 patients regardless of use of ventilation or not **(B)** Common genes are mostly involved in GO biological processes for cell division.

When comparing the genes of COVID-19 in those with and without ventilation, seven genes have been identified to be common between the two conditions (Figure 16A). These genes are expressed in COVID-19 regardless of the requirement for ventilation, hinting that they play a central role in COVID-19. Furthermore, gene enrichment analysis (Figure 16B) shows that these seven genes are involved with cytokinesis and cell division. Further anlysis through the COVID-19 reference lists reveal that these genes are still linked to cell division (Supplmentary Figure S7). An important factor to note is that most patients that require ventilation are those who are admitted to the ICU, this could be seen in the fourth analysis, where ICU patients that require ventilation, have more DEGs compared to the other conditions (Table 7). For example, ICU COVID-19 patients alone have more DEGs (431 genes) than non-ICU patients, the same for those that require ventilation (394 genes), which is more prominent when combining them both in the fourth analysis (Table 7).

**TABLE 9:** List of differentially expressed genes in GSE152641.

Upregulated in COVID-19	PPP1R14A, H2BC6, RRM2, IFI27, LY6E, H2AC4, SERPING1, IL22RA2, H3C8, FI44, ACOT2, RFFL, PARP6, H3C12, SPDYC, CNTNAP5, CLEC2L, DNTTIP1, IFNL4, RFPL4AL1, MED19, CHEK2, LRIT2, C12orf65, C1QC, MLNR, DBX2, RAB9B, PDZD8, GABRQ, NCKIPSD, ZFC3H1, CAV1, PPARG, ITGA7, LINC00460, HP, MINK1, MMP8, ZNRF1, LINC01442, IGFBP2, UCHL1, PAX3, KDM2B, SIGLEC1, RUNDC1, GPBAR1, LYRM4, OTOF, SDC1, PPP1R3G, C16orf87, SWI5, SPTLC3, ADAMTS2, OLFM4, CCL8, PRTN3, DEFA3, ADARB2
Down Regulated in COVID-19	FCER1A, OR10R2, GTPBP2, ALOX15, PACSIN2, SIGIRR, CHST9, TMEM97, GPR15, NOG, FDCSP, GSTM1, TUBB2A, GATD3A, TNS1, DISC1, SMIM13, HBG2

**FIGURE 17:**
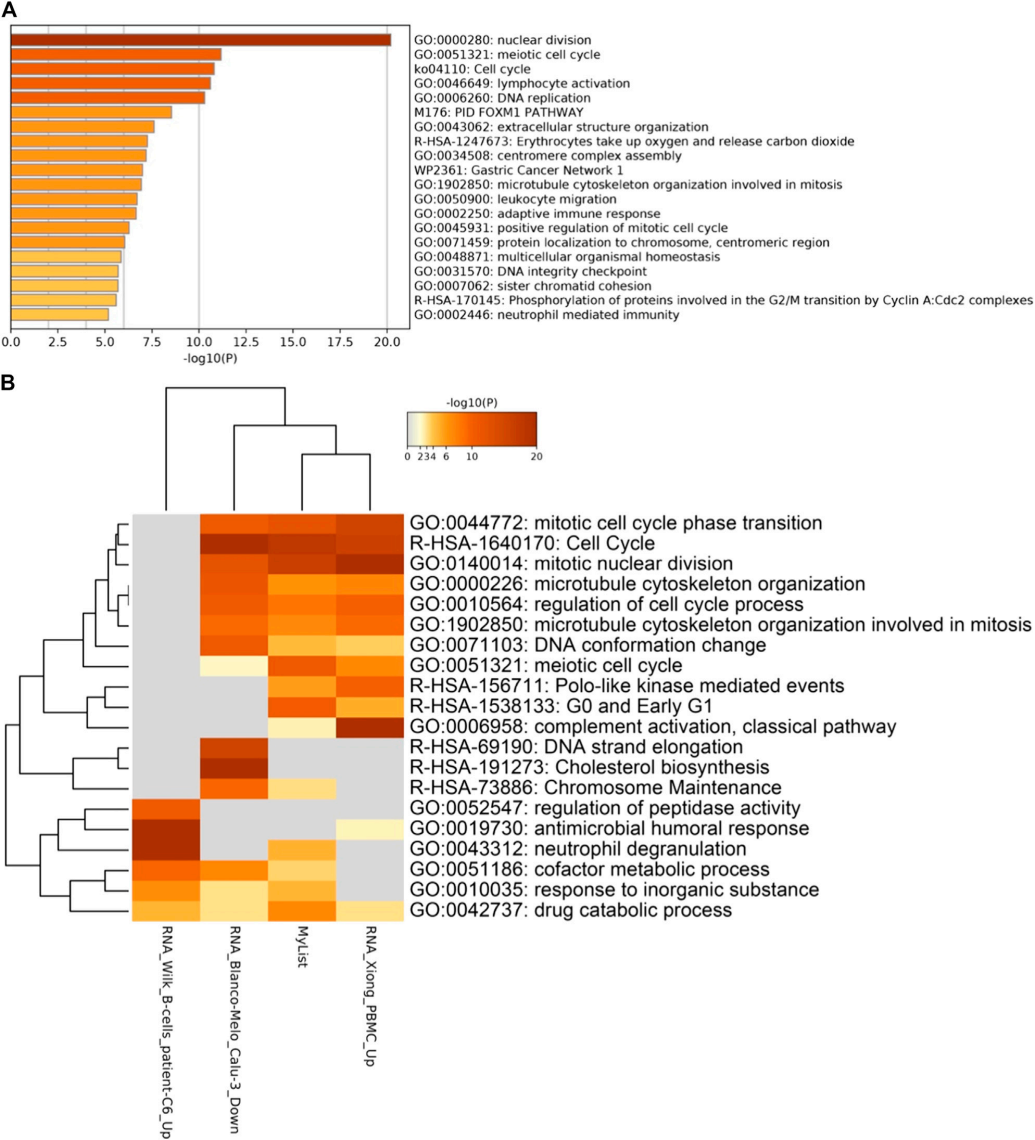
**(A)** enrichment analysis for the 387 ventilation only genes reveal most genes are involved in nuclear cell division. And **(B)** COVID-19 lists reveal that most genes are involved in the immune system.

Enriching the 387 COVID-19 ventilation specific genes (Figure 17A), revealed that most of the genes expressed are involved in cell division and cell cycle pathways. Comparing the DEGs to that of COVID-19 gene lists (Figure 17B), reveals the genes involved are those of the immune system pathways. This shows pathway enrichment analysis for genes yields different results than enrichment analysis specific for COVID-19.

As mentioned previously, the ICU genes of this data set were intersected with another dataset (GSE152418) and nine genes were found to be common between these two studies (Figure 15A).

### The Most Common Pathways Enriched Between Datasets Involve the Immune System and Its Regulation

Several pathways have been enriched in multiple datasets (Table 10), with cytokine signaling pathways and response to bacterium pathways being the most common. All of the enriched pathways involve the immune system, revealing a close link between viral infections and the immune response.

**TABLE 10:** List of pathways common between multiple datasets.

Pathway	Number of datasets pathway was enriched in
R-HSA-909733 Interferon alpha/beta signaling	2
R-HSA-1280215 Cytokine Signaling in Immune system	3
GO:0009617 response to bacterium	3
GO:0002274 myeloid leukocyte activation	2
GO:0035455 response to interferon-alpha	2
GO:0045055 regulated exocytosis	2
GO:0050900 leukocyte migration	2
M5885 NABA MATRISOME ASSOCIATED	2

However, in COVID-19 enriched pathways, there is a different set of genes and pathways that are enriched (Table 11).

**TABLE 11:** List of COVID-19 pathways common between multiple datasets.

Pathway	Number of datasets pathway was enriched in
GO:0002697 regulation of immune effector	2
GO:0001817 regulation of cytokine production	3
GO:0034341 response to interferon-gamma	3
R-HSA-1169410: Antiviral mechanism by INF-stimulated genes	2
GO:0009617 response to bacterium	4
R-HSA-1280215 Cytokine Signaling in Immune system	3
GO:0045088 regulation of innate immune response	2
GO:0035455 response to interferon-alpha	2
R-HSA-913531 Interferon Signaling	2
GO:0002683 negative regulation of immune system process	2
GO:0032103 positive regulation of response to external stimulus	2
GO:0050900 leukocyte migration	2
GO:0006898 receptor-mediated endocytosis	2
R-HSA-1474244: Extracellular matrix organization	2

The majority of COVID-19 enriched pathways are mostly involved in the regulatory aspect of the immune system. This sheds light to the viral mechanisms and how the virus affects the immune system, such as negatively regulating the immune response, one of the main causes for ARDS and disease progression. The most common pathways involved are those of the cytokines, more importantly interferons, which play a crucial role in SARS-CoV-2. Another pathway of interest is the response to bacterium pathway which is present in four different enriched lists from different datasets, indicating that perhaps COVID-19 could affect the microbiome. A clear link between these pathways can be seen as responding to the viral infections results into activation and subsequent regulation of the immune system and it’s various components. Studying the link between these pathways and the genes involved in them should give a better understanding of COVID-19 molecular basis and pathology.

## Conclusion

Using publicly available transcriptomic data we were able to identify differentially expressed genes in SARS-CoV-2 in multiple data sets (Figure 2). Of the nine data sets that were analyzed, only eight provided a list of differentially expressed genes. Using these lists of genes, the data was intersected and compared, where viable, and common genes between and within the datasets were found. However, each dataset contained a different number of genes in total, therefore the number of genes analyzed differened between datasets. Several genes have been present in several analyses as consistant DEGs, though the significance of their presence varied from analysis to another.

CRTAM has been identified as a gene that is present in COVID-19 patients, regardless of their comorbidities. Other genes such as CCRL2 and CCR6, even though belonging to the same family of chemokine, are differently regulated in SARS-CoV-2 infections. Furthermore, there seem to be several genes that are common between SARS-CoV-2 infection and other respiratory-causing viral infections such as MX1 which was also differentialy expressed in rotavirus infectoins. Furthermore, as the severity increases, the amount of shared genes increases, such as in GSE152418, all DEGs in severe patients were also expressed in ICU patients. Likewise, in GSE157103, when intersecting the DEGs for COVID-19 ICU with COVID-19 ventilation, there were many common genes between them.

An important note of observation is that the sample source played an important role in identifying differentially expressed genes. Such as when comparing gene expression from nasopharyngeal swabs with those from whole blood, the number of common genes of COVID-19 with other viral infections was higher in blood than in the nasal samples. However, there are 58 genes common between nasal swabs and whole blood, most of which are involved in antiviral responses. When comparing ARDS induced by either SARS-CoV-2, mechanical ventilation, or other respiratory illnesses, only five genes were identified as differentially expressed, one of which is involved with asthma and the other the immune system.

From this analysis, we can conclude that several genes appear to be less commonly expressed in different indications; however, their significance varies depending on the disease state and sample source. Some of the results of this analysis also validated some of the findings of the studies used. Further analysis is needed along with experimental validation to identify the potential biomarkers that could be used to characterize COVID-19 infections.

## Data Availability

The datasets presented in this study can be found in online repositories. The names of the repository/repositories and accession number(s) can be found in the article/Supplementary Material.
