# Supplementary material for: COVID-19 Transcriptomic Atlas: A Comprehensive Analysis of COVID-19 Related Transcriptomics Datasets
Source: Front Genet. 2021 Dec 22;12:755222. doi: 10.3389/fgene.2021.755222 (PMC8727884; doi:10.3389/fgene.2021.755222)
Supplement: Supplementary file 1 [file DataSheet1.docx]

**Supplementary Figures**

|  |
| --- |
| **Supplementary Figure 1.** Pathway Enrichment in COVID-19 lists for differentially expressed genes in COVID-19 induced ARDS. |

| 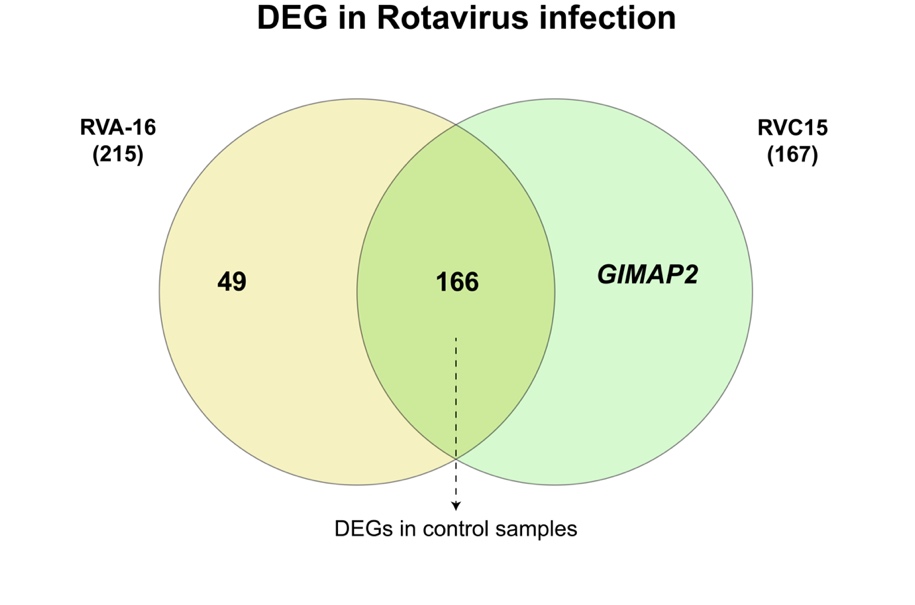 |
| --- |
| **Supplementary figure 2.** Differentially expressed genes in control, RVC-15 and RVA-16. |

| **** |
| --- |
| **Supplementary Figure 3.** COVID-19 pathway enrichment for the 58 genes that are common in COVID-19 patients between nasopharyngeal swabs and whole blood samples. |

|  |
| --- |
| **Supplementary Figure 4.** Gene enrichment for COVID-19 pathways from whole blood samples. |

| 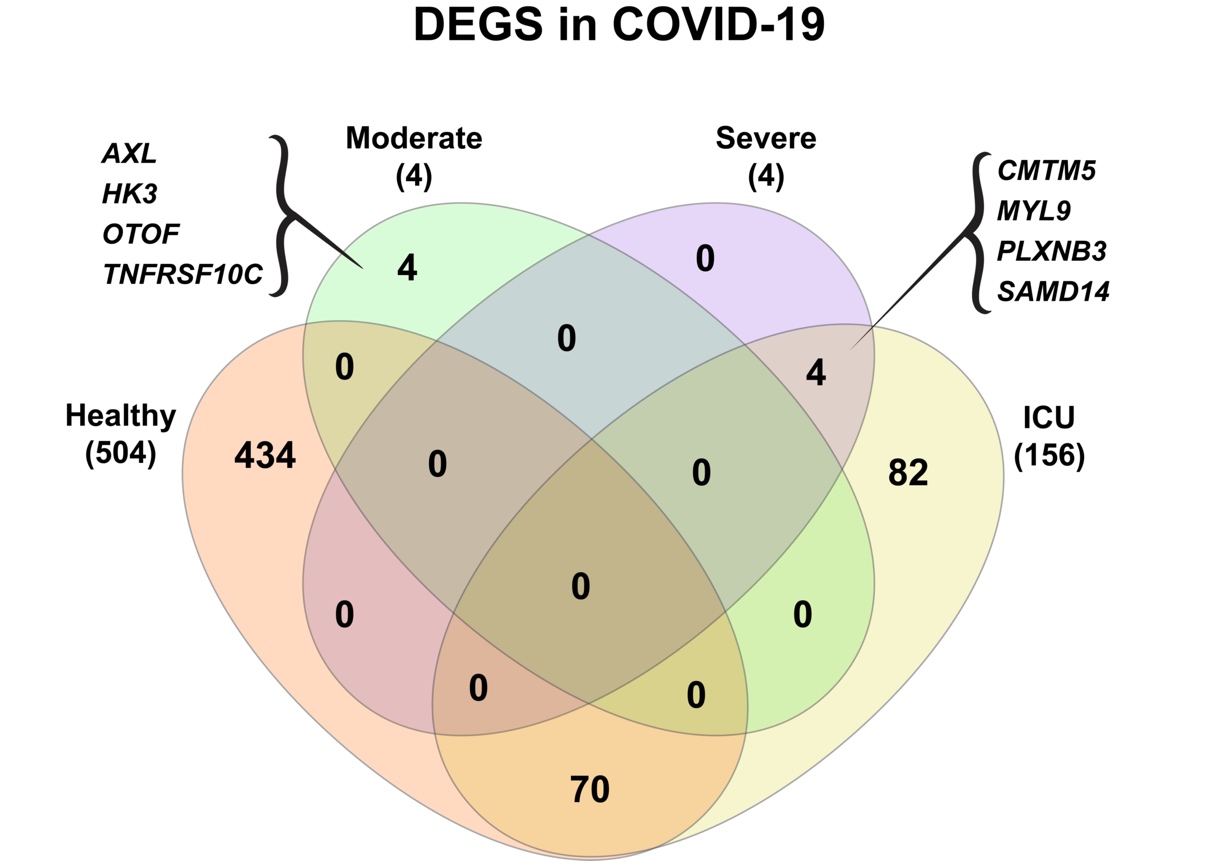 |
| --- |
| **Supplementary Figure 5.** Intersecting differential expressed genes based on disease state, with 4 genes common between severe and ICU patients, and 70 commons differentially expressed genes between healthy and ICU patients. Gene enrichment analysis shows that most genes are involved in hemostasis. |

|  |
| --- |
| **Supplementary Figure 6.** Only the ECM organization pathway is enriched in COVID-19 lists when comparing the common genes between ICU patients from different datasets. |

|  |
| --- |
| **Supplementary Figure 7.** Genes that are common between COVID-19 patients regardless of use of ventilation or not are involved in cell division in COVID-19 pathway enrichment |
